# Understanding Perceptions of Depression and Depression Care across Culture and Context

**DOI:** 10.3390/ijerph191811720

**Published:** 2022-09-17

**Authors:** Darrell Hudson, Akilah Collins-Anderson

**Affiliations:** Brown School, Washington University in St. Louis, St. Louis, MO 63130, USA

## 1. Introduction

Depression is a leading cause of disability, affecting approximately 300 million people globally [1]. In the United States, an estimated 8% of the adult population aged 20 or older will experience depression over an average 2-week period [2]. Heo and colleagues project that 46 million US adults will be diagnosed with a depressive disorder by 2050 [3]. Despite these alarming rates, there is still a great deal of research needed to fully understand the impact of depression. Marginalized populations experience significant barriers to depression care, including healthcare access barriers such as affordability and availability of care, which also contribute to underestimates of depression in these communities and unaddressed suffering [4,5,6]. Barriers also include the perceived appropriateness of care, especially if the modality of care is misaligned with consumers’ own preferences for care or the general norms around mental health within their social networks [6]. Extant data from the United States indicate that Black Americans have lower rates of depression compared to White Americans. However, there are nuances within this population. For example, rates appear to be different when scholars account for ethnicity and immigration experiences [7]. Additionally, some scholars have argued that the diagnostic tools used to measure depression symptoms and severity do not translate well across cultures, which could also lead to underestimates of depression among some racial/ethnic groups [8,9].

Similarly, notions of depression and depression care, including gender and contextual norms, vary across cultures [8,9]. Hegemonic norms of masculinity may affect the manifestation of depressive symptoms among men in addition to decreasing the likelihood of men seeking out depression care [10]. Norms of masculinity may also hinder providers’ ability to recognize depression among men [10]. It is also critical to examine depression from an intersectional perspective, simultaneously considering the role of different identity-related factors such race/ethnicity, gender, social class, immigration status, and sexual identity [Collins-Anderson et al., forthcoming]. For example, findings from a qualitative study of immigrants from sub-Saharan Africa who moved to the United States indicated that they were socialized to believe that mental health concerns indicated weakness or instability [7].

Additionally, the full impact of the COVID-19 pandemic, including the loss of loved ones, family conflict, job loss, and isolation due to transmission mitigation efforts in addition to overall uncertainty on depression has yet to be determined [11]. Nonetheless, it is likely that the incidence and burden of depression has significantly increased.

Notwithstanding the unknown impact of the pandemic on the prevalence and incidence of depression, there is a lack of up-to-date data on mental health. In the United States, the most recent national data on behavioral health prevalence were collected in the early 2000s via the Collaborative Psychiatric Epidemiologic Surveys (CPES) [12,13]. In the absence of up-to-date prevalence data, estimating depression needs and subsequent prevention and treatment programming is extremely difficult, especially for local municipalities and healthcare systems [14]. Alarmingly, fewer than half of the people diagnosed with major depressive disorder receive depression care, and among those who do receive treatment, only about 20% receive minimally adequate care [13]. Therefore, the impact of depression, such as its association with suicide, disability, and impairment, could be even greater than we currently estimate, especially when accounting for how perceptions of depression and depression care vary across cultures and contexts.

## 2. Conclusions

Taken together, it is critical to improve our understanding of depression and how to improve the provision of depression care. Furthermore, it is imperative to account for the effects of cultures and contexts on how depression as a condition is framed and how depression care is received. These are major challenges that span the globe. The papers in this issue of the *International Journal of Environmental Research and Public Health* (*IJERPH*) entitled “Perceptions of Depression and Depression Care” includes papers from scholars who are grappling with these questions in different contexts and across different cultures. 
